# Death following partner bereavement: A self-controlled case series analysis

**DOI:** 10.1371/journal.pone.0173870

**Published:** 2017-03-15

**Authors:** Michael King, Rebecca Lodwick, Rebecca Jones, Heather Whitaker, Irene Petersen

**Affiliations:** 1 Division of Psychiatry, University College London, London, United Kingdom; 2 Department of Primary Care and Population Health, University College London, London, United Kingdom; 3 Department of Mathematics and Statistics, The Open University, Milton Keynes, United Kingdom; Harvard Medical School, UNITED STATES

## Abstract

**Background:**

There is mixed evidence that older people bereaved of a spouse or partner are at risk of adverse outcomes. The main difficulty is to take account of other explanatory factors. We tested for an association between a patient’s death and the timing of any bereavement of a cohabitee.

**Method:**

Self-controlled case series study in which each case serves as his or her own control and which thereby accounts for all fixed measurable and unmeasurable confounders. We used the Health Improvement Network (THIN) primary care database to identify patients who died aged 50–99 years during the period 2003 to 2014. We used the household identifier in the database to determine whether they had an opposite sex cohabitee at the start of the observation period.

**Results:**

38,773 men and 23,396 women who had died and who had a cohabitee at the start of the observation period, were identified and included in male and female cohorts respectively. A higher risk of death was found in the 24 months after the death of the cohabitee than in the time classified as unexposed. The greatest risk was during the first 3 months after the death of the cohabitee (age-adjusted incidence rate ratio [IRR] 1.63, 95% CI 1.45–1.83 in the male cohort, and IRR 1.70, 95% CI 1.52–1.90 in the female cohort).

**Conclusion:**

Risk of death in men or women was significantly higher after the death of a cohabitee and this was greatest in the first three months of bereavement. We need more evidence on the effectiveness of interventions to reduce this increased mortality.

## Introduction

Grief is the constellation of psychological and physical reactions to the death of a spouse, relative, child or friend[1–3]. Bereavement is regarded as the most stressful of all life events[1] and bereaved older spouses and partners may be at risk of increased morbidity and mortality[3]. A recent systematic review and meta- analysis suggested that the mean hazard ratio (HR) was higher for bereaved men (HR, 1.27; 95% confidence interval (CI): 1.19, 1.35) than for women (HR: 1.15; 95% CI: 1.08, 1.22), with HRs decreasing more rapidly for men than for women as age increased[4]. It also seems that unexpected bereavement[5] and poorer economic circumstances[6] increase the risk. Possible reasons for elevated mortality have included emotional stress and its somatic consequences, homogamy as well as shared environmental risk factors such as smoking or diet, increased use of alcohol and recreational drugs, and poor self-care following bereavement. Good health and material circumstances do not seem to be protective[7] and lower access to health care does not make a major contribution to risk, at least in the US[6]. In the UK, however, there is evidence that reduced health care for cardiovascular disease before and after bereavement may play a role[8]. However, not all prospective studies agree on the findings[4, 9–11], possibly because of the difficulties of finding appropriate comparison cohorts and because of confounding. Research into whether death of a spouse or partner increases morbidity and mortality in older people has yielded conflicting results[4, 10, 11]. In an analysis of bereavement in primary care electronic records in the UK, we found little evidence for increased mortality[11] and a recent cohort study of older people in the US reported a similar lack of effect at least in bereaved women[12]. Thus, there appears to be an increased risk[4], but it is not a consistent finding. Residual confounding is a constant challenge as it is difficult to identify a suitable comparison group using standard cohort study design. For this reason we decided to apply the self-controlled case series method to this question.

Data from electronic health records are valuable for bereavement research as they contain data before bereavement, which is usually not available in a prospective study which recruits individuals. However, in all observational research we need study designs that reduce the risk of confounding. Self-controlled case series is a design in which each patient serves as their own control and which therefore implicitly accounts for fixed confounding factors. We used this design to address our main question, do people bereaved of a partner or spouse have an elevated risk of dying themselves? We examined primary care data on people who had died aged 50 and over to investigate whether risk of death was higher in the 24 months following bereavement from death of a cohabitee.

## Methods

### Data source

This study uses data from The Health Improvement Network (THIN) primary care database (http://www.csdmruk.imshealth.com). The THIN scheme for obtaining patient data and providing them in anonymised form to researchers was approved by the National Health Service South-East Multicentre Research Ethics Committee in 2002. The present study was approved by the University College London THIN steering committee and by the THIN scientific review committee (reference number: 15THIN062).

The THIN database contains data on over 11 million patients (including 3.7 million active patients) from over 500 general practices across the UK. Family doctors in the UK use the Read hierarchical coding system to record information on symptoms and diagnoses, and prescriptions[13, 14] are entered into the system automatically. The database also contains demographic information, and for patients no longer under active follow-up, the date of death or date that a patient has transferred out of the practice is recorded. A household identifier in the database indicates those patients who are living in the same household. The data used for this study were obtained from a license to THIN. For further information on access to the database, please contact IMS Health (contact details can be found at http://www.csdmruk.imshealth.com).

### Study design

This was a self-controlled case series study, a design which uses data only on individuals who have experienced the event of interest[15]. The SCCS method aims to estimate a relative incidence, which compares the incidence of adverse events within periods of hypothesised excess risk due to exposure with incidence during all other times. Asking “when?” rather than “who?” becomes the key question. For a description of and introduction to the self-controlled case series method see Petersen et al[16]. Hence, each person acts as their own control, so factors which do not change over time are implicitly accounted for. Included in this study were patients who had died, and the exposure was death of a cohabitee. Where the self-controlled case series method is used to analyse an outcome of death, information on exposure after the event of interest is typically censored at the death of the patient: in these circumstances, a modified method can be used that adjusts the incidence rate ratios for the unseen exposure time in the cases[17]. However, in our study, the exposure (cohabitee death) continues to be observed after the event (patient death). Consequently, we were able to use the standard self-controlled case series method.

### Study population

We identified patients who died aged 50–99 years during the period 2003 to 2014, and used the household identifier in the database to determine whether they had an opposite sex cohabitee at the start of the observation period. Patients were included in the analysis if there was only one other adult in the household (i.e. the cohabitee), and no more than four people aged under 18 years. This was to ensure that only those patients in individual households were included, as some residential homes or blocks of flats are identified in the database as households. Patients were excluded if the age gap with the cohabitee was greater than 15 years: this was to exclude situations where the person, who was identified as a cohabitee, was in fact a parent or adult child of the patient.

### Analysis

**Fig 1:**
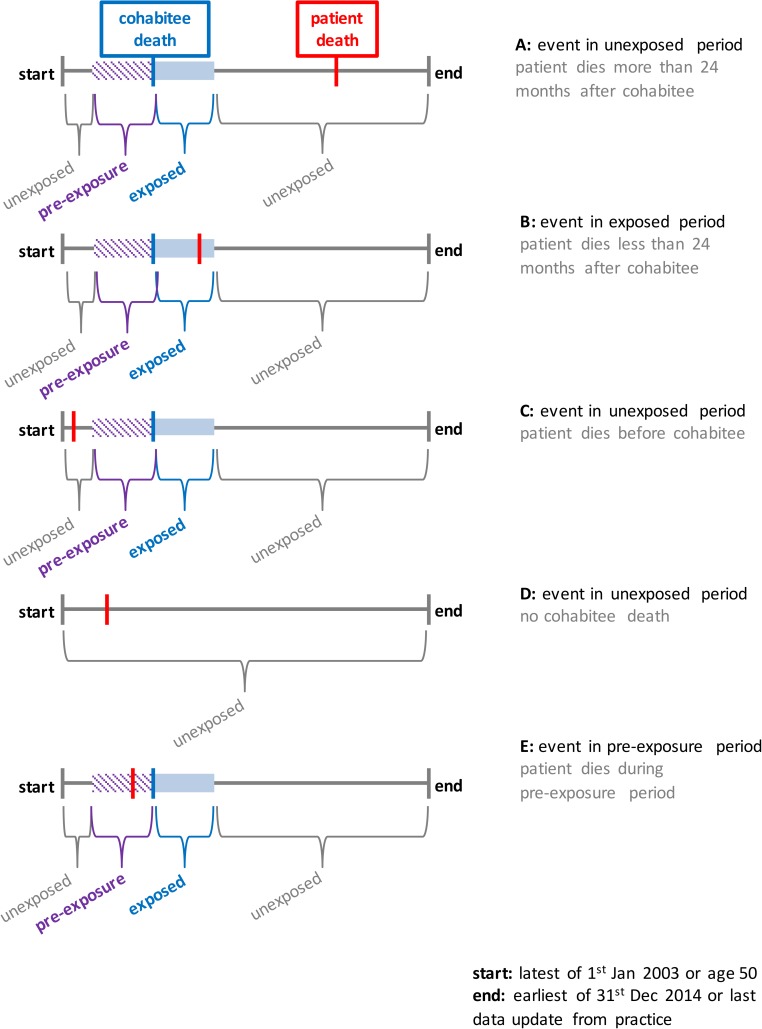
The study design and data scenarios.

In this analysis, the event was patient death and the exposure was cohabitee death, both of which could occur at any point in the observation period (Fig 1). The start of the observation period was the latest of 1^st^ January 2003 or the date on which the patient was aged 50, and the end of the observation period was the earliest of 31^st^ December 2014 or the last date for which practice data were available. Only those patients who had died during the observation period were included. The risk period associated with the exposure was defined as 24 months after cohabitee death: this was divided into eight risk intervals, each of three months’ duration.

We performed separate analyses for male patients (with female cohabitee deaths as exposures) and for female patients (with male cohabitee deaths as exposures). Given we included only opposite sex patient-cohabitee pairs, no individual was included both as a patient and as a cohabitee in the same analysis. Thus, there was a clear *a priori* definition of which household deaths were viewed as events and exposures in each analysis.

One of the underlying assumptions of the self-controlled case series approach is that occurrence of the event (patient death) does not affect the subsequent exposure (cohabitee death). If, for example, there exists an inflated risk of male patient death following female cohabitee death, it follows that there may similarly be an inflated risk of female cohabitee death following male patient death. To correct for any bias arising as a result of this reverse causation, we included a pre-exposure risk period of 24 months before cohabitee death[16, 18]. Time that the patient spent unexposed, used as the reference in the analysis, was therefore time prior to the pre-exposure risk period, or from 24 months after cohabitee death. Where the cohabitee was still living at the end of the follow-up period, all time included for that patient was unexposed (Fig 1).

Possible differences by pre-death illness and cause of death of the cohabitee were investigated in supplementary analyses by restricting the definition of exposure to: (i) death of a cohabitee with dementia; and (ii) death of a cohabitee who had a cancer diagnosis in the previous five years. A sensitivity analysis was performed where the inclusion criteria for households were relaxed so that, in addition to the patient and the cohabitee, a third adult was allowed to be in the same household: either an older person (at least 20 years older than both the patient and cohabitee), or a younger adult (aged 18–30, and at least 20 years younger than both the patient and cohabitee).

Each patient served as their own control in this analysis, which thereby accounted for fixed confounding factors. The incidence rate ratios were adjusted for age, which was modelled as a categorical variable consisting of a number of narrow age bands: 50–59, 60–69, 70–74, 75–79, 80–84, 85–89, 90–94, and 95–99.

## Results

A total of 38,773 men and 23,396 women who had died aged 50 to 99 between 2003 and 2014, and who had an opposite sex cohabitee at the start of the observation period, were identified and included in the male and female cohorts respectively. The median age at death was 80 years in the male cohort and 79 years in the female cohort. Cohabitee death occurred before patient death, either during or after the risk period (Fig 1A or 1B), for 4,164 (10.7%) of patients in the male and 4/954 (21.2%) of patients in the female cohort (Table 1).

**Table 1:** Characteristics of included patients, United Kingdom, 2003–2014.

	Male patients	Female patients
	(female cohabitees)	(male cohabitees)
Number of patients	38,773	23,396
Age of patient at index date–*median (IQR)*	75 (69–80)	74 (67–80)
Age of patient at death–*median (IQR)*	80 (74–85)	79 (72–85)
**Cohabitee status**		
cohabitee death before patient death (Fig 1A or 1B)	4,164 (10.7%)	4,954 (21.2%)
cohabitee death after patient death (Fig 1C or 1E)	5,004 (12.9%)	4,217 (18.0%)
cohabitee still living (Fig 1D)	29,605 (76.4%)	14,225 (60.8%)

IQR = interquartile range.

**Fig 2:**
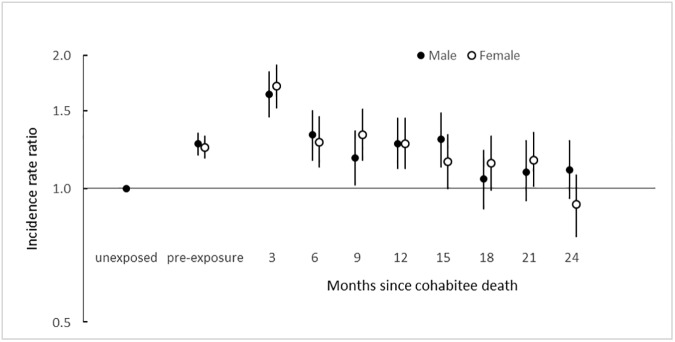
Incidence rate ratios from a self-controlled case series analysis for patient death during the 24 months after cohabitee death.

In the male cohort, 1,701 (4.4%) of patient deaths occurred during 24 months after the death of the female cohabitee. In the female cohort, 1,783 (7.6%) of patient deaths occurred during the 24 months after the death of the male cohabitee. For both male and female patients, the risk of death was highest immediately after the death of the opposite sex cohabitee, and fell over the next 24 months to the unexposed level (Table 2, Fig 2). The greatest risk was observed during the first three months after the death of the cohabitee, with age-adjusted incidence rate ratio (IRR) 1.63 (95% CI: 1.45, 1.83) in the male cohort, and IRR 1.70 (95% CI: 1.52, 1.90) in the female cohort (Table 2). A raised risk of patient death, compared to the unexposed time, was observed during the pre-exposure period of 24 months before cohabitee death.

**Table 2:** Incidence rate ratios (IRR) for patient death by risk period.

	Male patients			Female patients		
	*exposure*: *death of female cohabitee*			*exposure*: *death of male cohabitee*		
	IRR	95% CI	Events	IRR	95% CI	Events
Time since cohabitee death						
unexposed	1	-	35,304	1	-	19,923
pre-exposure period	1.26	1.19 to 1.33	1,768	1.24	1.17 to 1.31	1,690
3 months	1.63	1.45 to 1.83	306	1.70	1.52 to 1.90	322
6 months	1.32	1.16 to 1.50	238	1.27	1.12 to 1.45	235
9 months	1.17	1.02 to 1.35	205	1.32	1.16 to 1.51	240
12 months	1.26	1.11 to 1.44	227	1.26	1.11 to 1.44	237
15 months	1.29	1.12 to 1.48	212	1.15	1.00 to 1.32	200
18 months	1.05	0.90 to 1.22	168	1.14	0.99 to 1.31	195
21 months	1.09	0.94 to 1.28	170	1.16	1.01 to 1.34	195
24 months	1.10	0.95 to 1.28	175	0.92	0.78 to 1.07	159

IRR = incidence rate ratio; CI = confidence interval.

Defining exposure as death of a cohabitee with a record of dementia reduced the number of patient deaths classed as occurring during the risk period, with 177 (0.5%) deaths in the male and 143 (0.6%) deaths in the female cohort occurring during the 24 months risk period after cohabitee death. The risk of patient death in the first three months after cohabitee death with dementia was significantly higher than during the unexposed period in both the male and female cohorts (male cohort IRR: 2.08; 95% CI: 1.47, 2.95; female cohort IRR: 1.64 95% CI: 1.07, 2.52; Table 3). Defining instead exposure as death of a cohabitee who had a record of cancer in the previous five years resulted in 527 (1.4%) patient deaths in the male and 654 (2.8%) patient deaths in the female cohort occurring during the risk period of 24 months after cohabitee death. The risk of patient death was significantly higher in the three months after cohabitee death with cancer compared to the unexposed time (male cohort IRR: 1.48; 95% CI: 1.19, 1.83; female cohort IRR: 1.72; 95% CI: 1.43, 2.07; Table 3).

**Table 3:** Summary of results from supplementary analyses.

		Dementia		Cancer		Household	
		*exposure*: *death of a cohabitee with a record of dementia*		*exposure*: *death of a cohabitee with a record of cancer in previous five years*		*expanded definition of eligible household to allow an additional older person or younger adult*	
Cohort	Time since cohabitee death	IRR	95% CI	IRR	95% CI	IRR	95% CI
**Male**	unexposed	1	-	1	-	1	-
	pre-exposure period	1.06	0.88 to 1.29	1.26	1.15 to 1.39	1.25	1.19 to 1.32
	3 months	2.08	1.47 to 2.95	1.48	1.19 to 1.83	1.63	1.46 to 1.83
	6 months	1.92	1.32 to 2.77	1.48	1.19 to 1.84	1.31	1.15 to 1.49
	9 months	1.20	0.75 to 1.92	1.14	0.89 to 1.47	1.16	1.01 to 1.33
	12 months	1.58	1.05 to 2.38	1.08	0.83 to 1.39	1.26	1.10 to 1.44
	15 months	1.60	1.04 to 2.46	1.09	0.84 to 1.42	1.28	1.12 to 1.47
	18 months	1.29	0.79 to 2.09	1.26	0.98 to 1.61	1.05	0.90 to 1.23
	21 months	1.26	0.76 to 2.08	1.05	0.80 to 1.38	1.09	0.94 to 1.27
	24 months	1.25	0.76 to 2.06	1.17	0.90 to 1.51	1.10	0.95 to 1.28
**Female**	unexposed	1	-	1	-	1	-
	pre-exposure period	1.06	0.85 to 1.31	1.17	1.07 to 1.29	1.24	1.17 to 1.31
	3 months	1.64	1.07 to 2.52	1.72	1.43 to 2.07	1.69	1.51 to 1.89
	6 months	1.33	0.82 to 2.16	1.24	0.99 to 1.55	1.27	1.12 to 1.45
	9 months	1.77	1.15 to 2.72	1.23	0.98 to 1.54	1.32	1.16 to 1.50
	12 months	1.82	1.19 to 2.77	1.32	1.07 to 1.63	1.27	1.11 to 1.44
	15 months	1.11	0.64 to 1.94	1.32	1.06 to 1.65	1.14	0.99 to 1.31
	18 months	1.41	0.85 to 2.32	1.07	0.84 to 1.37	1.13	0.98 to 1.31
	21 months	1.98	1.29 to 3.05	1.37	1.10 to 1.71	1.15	1.00 to 1.33
	24 months	0.70	0.35 to 1.41	0.86	0.66 to 1.13	0.91	0.78 to 1.07

IRR = incidence rate ratio; CI = confidence interval. The risk period was time from cohabitee death up to 24 months after cohabitee death. IRR are relative to time outside the risk periods, and are adjusted for age.

Expanding the definition of an eligible household by allowing for either an older person or younger adult in addition to the patient and cohabitee increased the number of patients included in the analysis to 39,288 for the male cohort and 23,700 for the female cohort (an increase of 1.3% in both cohorts), and gave similar IRR estimates to those in the main analysis (Table 3).

## Discussion

### Findings

Our main finding was that, after adjustment for age, risk of death in men or women was significantly higher following the death of a cohabitee, and that this was greatest in the first three months of bereavement. This elevated risk was similar when our analyses focused on bereavement following deaths from dementia or cancer, with a somewhat higher risk following the former. When we relaxed our entry criteria and included households which also contained an older or younger adult in addition to the patient and cohabitee, the numbers of patients included changed very little and our results were essentially unchanged. At first sight it may seem counter-intuitive that the median age of death of men in our population was slightly higher than that of women, as male life expectancy is usually lower than female. This occurred because the method required them to be co-habitants. If all patients in this age group had been involved (cohabiting or living alone) the age profile would be as expected. It may also seem counter-intuitive that risk of death appears highest at ages 80–84 and then seems to decrease with older age. This reflects the age distribution in our selected population, with a median age at death around 80.

### Strengths and limitations

Using electronic primary care health records allowed us to include data on a large number of bereaved individuals, a group for which study recruitment is challenging, and for whom there was data over years before the bereavement. The self-controlled case series method ensured that all fixed confounders (measureable and unmeasurable) are accounted for. We took account of the assumption in a self-controlled case series that occurrence of the event does not affect subsequent exposure by removing from the unexposed time a pre-exposure risk period of 24 months before the cohabitee death. However, given age is the strongest risk factor for death and is time varying we needed to adjust for it in the analysis. A limitation of this study is that, although we presumed most were partner relationships, we do not know the true nature of the relationship between the patient and the cohabitee. To avoid non-partner relationships, we excluded patients in large households from the analysis and included only those patient-cohabitee pairs aged within 15 years of each other. Our sensitivity analysis in which we relaxed the criteria on other adults living in the household did not alter the numbers or results to any degree.

### Implications

Our results add to the growing body of evidence that when one member of a couple dies, the one left is at greater risk of dying especially in the first three months following their bereavement[5]. An actuarial country wide Norwegian study of married couples reported similar risks but especially occurring in the first seven days[19] and in the younger-old. A particularly high risk occurred on the same day but that may simply be that both members of a couple dies in the same incident. A similar elevation in risk is seen in parents bereaved of a child[20]. Given that neither material circumstances, nor access to health care, appear to be protective[6, 7], psychological and lifestyle factors may well play a key role. Our earlier analyses using the THIN database demonstrated that bereaved widows and widowers were about twice as likely to be prescribed hypnotics or antidepressants, even after adjustment for levels of such prescribing before the partner’s death[11]. It has long been thought that the emotional stress of bereavement might be part of the mechanism of increased mortality in bereavement[21] through changes in pulse rate, arterial blood pressure and endocrine function. This may be compounded by neglect of cardiovascular health care both before and after the bereavement[8]. The finding that an unexpected death elevates the risk even further would support this conclusion[5]. Important mechanisms that may mediate the pathway from bereavement to increased mortality are changes in finance and social support, poor sleep which possibly leads to greater use of psychotropic drugs and alcohol, and poor self-care particularly nutritional neglect. A recent cohort study appears to show that depression mediates the relationship between bereavement and subsequent mortality most particularly in men[12].

Grief counselling in the early months of bereavement, together with a more specific talking therapy for those with complicated grief, might play the greatest role in reducing this risk. However, evidence on the effectiveness of grief counselling is weak, mainly because of a lack of well-designed effectiveness research. There is also uncertainty about the rationale for the counselling approach employed, including when to offer it and to whom[22]. We also need a more consistent approach to management of grief in primary medical care. Calls for bereavement protocols in general practice have been made for at least 20 years[23] but we still lack evidence on their effectiveness or extent of implementation. We need to know more about how and when to intervene after bereavement, as well as the effectiveness of interventions to reduce the immediate stress of grief, and the increased mortality and morbidity that occurs in the longer term.

## References

[1] Woof WR, Carter YH. The grieving adult and the general practitioner: a literature review in two parts (Part 1). Br J Gen Pract. 1997;47(420):443–8. 9281874 PMC1313057

[2] Parkes CM. Bereavement in adult life. BMJ. 1998;316(7134):856–9. 9549464 10.1136/bmj.316.7134.856 PMC1112778

[3] Stroebe M, Schut H, Stroebe W. Health outcomes of bereavement. Lancet. 2007;370(9603):1960–73. 10.1016/S0140-6736(07)61816-9 18068517

[4] Shor E, Roelfs DJ, Curreli M, Clemow L, Burg MM, Schwartz JE. Widowhood and Mortality: A Meta-Analysis and Meta-Regression. Demography. 2012;49(2):575–606. 10.1007/s13524-012-0096-x 22427278 PMC3640496

[5] Shah SM, Carey IM, Harris T, DeWilde S, Victor CR, Cook DG. The Effect of Unexpected Bereavement on Mortality in Older Couples. American Journal of Public Health. 2013;103(6):1140–5. 10.2105/AJPH.2012.301050 23597341 PMC3670660

[6] Simeonova E. Marriage, bereavement and mortality: The role of health care utilization. Journal of Health Economics. 2013;32(1):33–50. 10.1016/j.jhealeco.2012.10.010 23202255

[7] Shah SM, Carey IM, Harris T, DeWilde S, Victor CR, Cook DG. Do Good Health and Material Circumstances Protect Older People From the Increased Risk of Death After Bereavement? American Journal of Epidemiology. 2012;176(8):689–98. 10.1093/aje/kws162 23051600 PMC3472615

[8] Shah SM, Carey IM, Harris T, DeWilde S, Victor CR, Cook DG. The Impact of Partner Bereavement on Quality of Cardiovascular Disease Management. Circulation. 2013. 10.1161/CIRCULATIONAHA.113.004122 24255060

[9] Ebrahim S, Wannamethee G, McCallum A, Walker M, Shaper AG. Marital status, change in marital status, and mortality in middle-aged British men. American Journal of Epidemiology. 1995;142(8):834–42. 7572960 10.1093/oxfordjournals.aje.a117723

[10] Schaefer C, Quesenberry CP, Soora W. Mortality following conjugal bereavement and the effects of a shared environment. American Journal of Epidemiology. 1995;141:1142–52. 7771452 10.1093/oxfordjournals.aje.a117387

[11] King M, Vasanthan M, Petersen I, L J, Marston L, Nazareth I. Medical Care after Bereavement: A General Practice Cohort Study. PLOS One. 2013;8(1). 10.1371/journal.pone.0052561 PMC3556004 23372651

[12] Stahl ST, Arnold AM, Chen J-Y, Anderson S, Schulz R. Mortality After Bereavement: The Role of Cardiovascular Disease and Depression. Psychosom Med. 2016;78(6):697–703. 10.1097/PSY.0000000000000317 26894326 PMC4927386

[13] Booth N. What are the Read Codes? Health Libraries Review. 1994;11(3):177–82. 10139676 10.1046/j.1365-2532.1994.1130177.x

[14] Dave S, Petersen I. Creating medical and drug code lists to identify cases in primary care databases. PharmacoepidemiolDrug Saf. 2009;18(8):704–7. 10.1002/pds.1770 19455565

[15] Whitaker HJ, Farrington CP, Spiessens B, Musonda P. Tutorial in biostatistics: the self-controlled case series method. Statistics in Medicine. 2006;25(10):1768–97. 10.1002/sim.2302 16220518

[16] Petersen I, Douglas I, Whitaker H. Self controlled case series methods: an alternative to standard epidemiological study designs. BMJ. 2016;354. 10.1136/bmj.i4515 27618829

[17] Farrington CP, Whitaker HJ, Hocine MN. Case series analysis for censored, perturbed, or curtailed post-event exposures. Biostatistics. 2009;10(1):3–16. 10.1093/biostatistics/kxn013 18499654

[18] Farrington CP, Whitaker HJ. Semiparametric analysis of case series data. Journal of the Royal Statistical Society: Series C (Applied Statistics). 2006;55(5):553–94.

[19] Ytterstad E, Brenn T. Mortality After the Death of a Spouse in Norway. Epidemiology. 2015;26(3):289–94. 10.1097/EDE.0000000000000266 25695353

[20] Schorr L, Burger A, Hochner H, Calderon R, Manor O, Friedlander Y, et al Mortality, cancer incidence, and survival in parents after bereavement. Annals of Epidemiology. 2016;26(2):115–21. 10.1016/j.annepidem.2015.12.008 26809234

[21] Parkes CM, Benjamin B, Fitzgerald RG. Broken Heart: A Statistical Study of Increased Mortality among Widowers. British Medical Journal. 1969;1(5646):740–3. 5769860 10.1136/bmj.1.5646.740 PMC1982801

[22] Waller A, Turon H, Mansfield E, Clark K, Hobden B, Sanson-Fisher R. Assisting the bereaved: A systematic review of the evidence for grief counselling. Palliative Medicine. 2015. 10.1177/0269216315588728 26415735

[23] Charlton R, Dolman E. Bereavement: a protocol for primary care. The British journal of general practice: the journal of the Royal College of General Practitioners. 1995;45(397):427–30. Epub 1995/08/01. PubMed Central PMCID: PMCPMC1239338. 7576849 PMC1239338

